# Tobacco smoke and all-cause mortality and premature death in China: a cohort study

**DOI:** 10.1186/s12889-023-17421-w

**Published:** 2023-12-12

**Authors:** Liang Zhang, Yonghong Ma, Ke Men, Chao Li, Zhuo Zhang, Guoshuai Shi

**Affiliations:** 1 Cardio-Aortic surgery center, AnHui Chest Hospital, Hefei, Anhui China; 2 School of Public Health, Xi’an Medical College, Xi’an, Shaanxi China; 3 Research Center for Medical Prevention and Control of Public Safety of Shaanxi Province, Xi’an, Shaanxi China; 4 Department of Epidemiology and Biostatistics, Xi’an Jiaotong University Health Science Center, Xi’an, Shaanxi China; 5 School of Health Services Management, Xi’an Medical College, Xi’an, China

**Keywords:** Tobacco smoke, Mortality, Premature death, Cohort study

## Abstract

**Background:**

Tobacco smoke is associated with several diseases, and identified as the second leading risk factor for death from any cause worldwide. The relationship of tobacco smoke to mortality or premature death is not yet available from contemporary cohorts after 2010 in China. This study aimed to investigate the smoking behavior and the relationship of tobacco smoke to mortality and premature death among a nationally representative cohort starting from 2011 in China.

**Methods:**

The nationally representative datasets (China Health and Retirement Longitudinal Study, CHARLS, 2011–2012) was employed and linked with follow-up data (2013). CHARLS was an ongoing nationally representative survey, which longitudinally followed up subjects aged over 45 years. Smoking status (non-smoker, ex-smoker, smoker, pack-years of smoking, age at starting and ceasing smoking) was used as independent variable, and all-cause mortality, premature death (defined as mortality before age 72.7 years in men and 76.9 years in women) were used as dependent variables. The Cox’s proportional hazards regression mode was used to estimate the effect of tobacco smoke and pack-years of smoking on all-cause mortality and premature death.

**Results:**

A total of 16,701 subjects were included. The association between tobacco smoker (hazard ratio [HR] = 1.37, 95%CI = 1.02, 1.83) / ex-smoker (HR = 1.75, 95%CI = 1.24, 2.46) and all-cause mortality was significant. Tobacco smoker (HR = 1.58, 95%CI = 1.04, 2.39) and ex-smoker (HR = 2.25, 95%CI = 1.38, 3.66) was associated with increase in the risk of premature death. Pack-years of smoking ≥ 30 was associated with increased risk of premature death compared with non-smokers in total (HR = 1.59, 95%CI = 1.03, 2.43) and women (HR = 3.38, 95%CI = 1.22, 9.38). Additionally, our results also revealed that there was a linear trend between pack-years of smoking and premature death in total (P = 0.002) and women (P = 0.010).

**Conclusion:**

This study found a negative effect of smoking status on all-cause mortality and premature death among a contemporary and nationally representative data in China. The correlation between pack-years of smoking and premature death and the trend of pack-years of smoking with premature death was also identified.

**Supplementary Information:**

The online version contains supplementary material available at 10.1186/s12889-023-17421-w.

## Background

Tobacco smoke has been identified as the second leading risk factor for mortality on a global scale [1, 2]. The World Health Organization (WHO) has projected that in 2006, approximately 5.4 million individuals worldwide succumbed to tobacco-related illnesses, and unless immediate measures are implemented, the annual death toll attributable to tobacco is expected to surpass eight million by 2030 [3]. It is estimated that between 2002 and 2030, deaths attributed to tobacco will decline by 9% in developed nations, while experiencing a 100% increase (reaching 6.8 million) in developing countries [4].

To the best of our knowledge, there is a growing body of large-scale quantitative evidence regarding the association between tobacco smoke and mortality or premature death in countries experiencing a mature smoke epidemic. This evidence includes findings from the British Doctors Study [5] and the American Cancer Society Cancer Prevention Study [6]. However, there is currently a lack of contemporary cohort studies conducted after 2010 in China that provide such evidence. Additionally, it is important to note that China has one of the highest prevalence of tobacco use among men (50.5% among men aged 15 years and over). This translates to over 300 million smokers and 740 million non-smokers being exposed to second-hand smoke [7]. A longitudinal study spanning 15 years revealed that both current and former smokers in rural China exhibited an elevated susceptibility to mortality. Additionally, the investigation established a correlation between pack-years and the age at starting smoking with overall mortality rates [8]. Consequently, the data derived from China’s context are anticipated to furnish valuable perspectives on the hazards posed by tobacco smoke in regions characterized by a high prevalence of smoking.

The objective of this study was to examine the smoking habits and the relationship of tobacco smoke to mortality and premature death among a nationally representative cohort in China, starting from 2011. The outcomes of this research have the potential to enhance global understanding by offering supplementary independent evidence on the consequences of long-term, heavy, and widespread smoking.

## Methods

### Study population and design

In this study, we utilized the national baseline database of the China Health and Retirement Longitudinal Study (CHARLS). The primary objective of CHARLS was to establish a high-quality public micro-database, encompassing an extensive array of information serving the demands of scientific inquiry and policy formulation on ageing-related matters. The study design for CHARLS had been reported previously [9, 10]. In general, it was an ongoing nationally representative survey, which longitudinally follows up people aged over 45 years in China (follow-up survey performed every 2 years for a total of 3 waves from 2013 to 2018). CHARLS national baseline survey was conducted in 2011–2012 using the multi-stage probability to proportional to size (PPS) sampling method. The samples covered 450 villages, 150 counties, and 28 provinces, involving more than 17,708 people from about 10 000 households and could be weighted to obtain national estimates. Participants enrolled in baseline survey were followed up in waves 2, 3 and 4. Both interview status (dead or alive) and death time were recorded in wave 2 (2013 follow-up). For waves 3 and 4, only the interview status information was available. Detailed information on demographic characteristics, medical history, prescription drug use, clinical measurement and laboratory testing was collected using a standard questionnaire.

### Variable definition

#### Demographic characteristics

We obtained information on demographic characteristics (age, educational level [illiterate, Primary, Secondary/High School, College/University or above], sex, residence status [Rural, Urban], marital status [Married/ Divorced/ Widowed, Unmarried]).

#### Hypertension, diabetes, dyslipidemia and cardiovascular disease (CVD)

We calculated the average systolic blood pressure (SBP) and diastolic blood pressure (DBP) based on 3 blood pressure measurements (approximately 45s apart) for each subject (Omron model HEM-7200). Hypertension was defined as SBP ≥ 140 mm Hg, or DBP ≥ 90 mm Hg, or self-reported utilization of antihypertensive treatment. Diabetes was defined as hemoglobin A_1C_ ≥ 6.5%, utilization of diabetes medication, or self-reported history of diabetes. Dyslipidemia was defined as utilization of dyslipidemia medication or meeting one of the following conditions: total cholesterol ≥ 6.19 mmol/L, low density lipoprotein cholesterol ≥ 4.14 mmol/L, or triglyceride ≥ 2.27 mmol/L. History of CVD was defined as self-reported medical history (i.e., history of stroke; history of heart failure; history of coronary heart disease, defined as angina, myocardial infarction or reported coronary heart disease).

#### Smoking status, drinking status and overweight/obesity

Smoking status included non-smoker, ex-smoker, smoker, smoking duration, Cigarettes consumed per day, pack-years (number of years of smoke*number of cigarettes consumed per day/20) and age at starting and ceasing smoking). Drinking status included drinker and non-drinker. BMI was calculated by the square of weight/height and overweight and obesity was defined as BMI ≥ 24 kg/m^2^ [11].

#### Premature death

The date of all-cause mortality was only available during the 2013 follow-up survey, so the age of death was calculated as the interval between the date of birth in baseline information and the death date in 2013 follow-up survey. Premature death was defined as mortality before 72.7 years for men and 76.9 years for women, which was average life expectancy in China in 2011 [12]. Detailed information about data quality management had been previously estimated [13].

### Statistical analysis

Summary statistics for the prevalence of smokers, smoking status and baseline characteristics by sex were computed as proportions or means. The effect of tobacco smoke and pack-years of smoking on all-cause mortality and premature death were estimated using Cox’s proportional hazards regression model. Unadjusted and adjusted hazard ratios (HRs) and their 95%CIs were obtained. The multivariable model was adjusted for sex, age, educational level, current residence status, marital status, hypertension, dyslipidemia, diabetes, history of CVD, drinking status, overweight or obesity. P values for trend were calculated using the quartile median values. All reported P values were 2-tailed, and values of α < 0.05 were considered statistically significant. SAS software version 9.4 was used for analysis.

## Results

In the present study, we excluded 5.7% (1007/17,708) participants, because of the missing values in age/sex (n = 70), current smoking (n = 126), ceasing smoking (n = 652) and other smoking details (n = 159). Therefore, the remaining 16,701 participants were eligible for inclusion in the present analysis and 367 deaths were followed up.

**Fig. 1:**
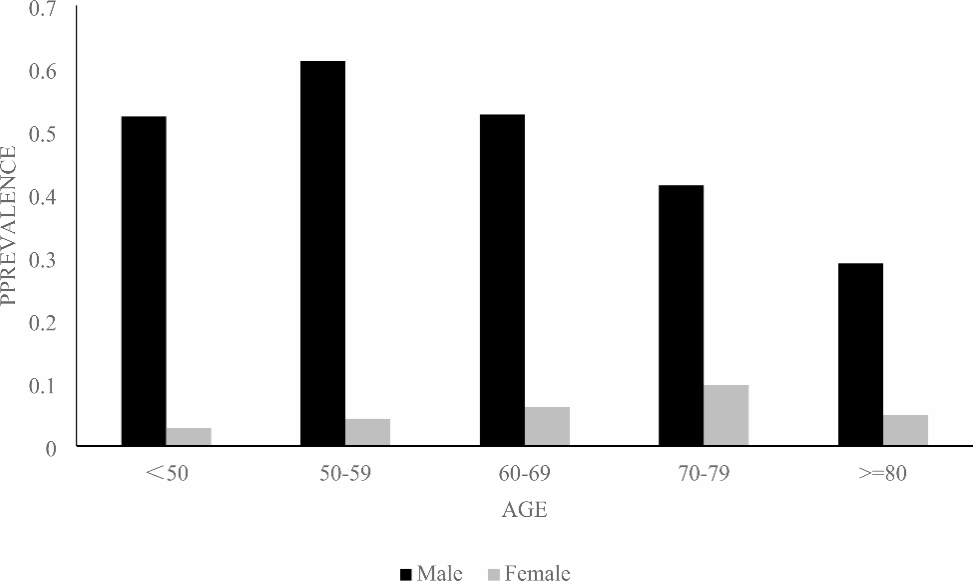
Prevalence of tobacco smoke by age and sex

The estimated prevalence of current smoker aged 45 and over ranges from 29.03% (≥ 80 years group) to 52.68% (60–69 years group) among men, and from 2.84% (45–49 years group) to 9.72% (70–79 years group) among women (Fig. 1). The smoking status by sex were shown in Table 1. The prevalence of smokers was higher in men (55.91%). Moreover, men were likely to have longer smoking duration (mean: 35.2 years; mainly from 20 to 39 years’ group), consume more cigarettes per day (mean: 19.6; concentrated on 20–39 group), start smoke at younger age (mean: 22.2 years; mainly from 18 to 25 years’ group). The age at ceasing smoking was similar between men and women (52.1 vs. 52.3 years old).

**Table 1:** Smoking Status among smoker and non-smoker, by sex

	Men (N = 7600)	Women (N = 8681)
Non-smoker	2194 (28.65)	8362(92.46)^1^
Ex-smoker	1182 (15.44)	164 (1.81)^1^
Smoker	4281 (55.91)	518 (5.73)^1^
Smoking duration (years)		
Mean (SD)	35.2 (12.4)	32.8 (16.8)^1^
< 20	514 (9.41)	155 (22.73)^1^
20–39	3002 (54.95)	280 (41.06)
≥ 40	1947 (35.64)	247 (36.22)
Cigarettes/day		
Mean (SD)	19.6 (12.6)	12.6 (9.6)^1^
< 20	1518 (36.38)	250 (65.27)^1^
20–39	2100 (50.32)	120 (31.33)
≥ 40	555 (13.30)	13 (3.39)
Age at starting smoking (years)		
Mean (SD)	22.2 (8.0)	26.3 (13.2)^1^
< 13	196 (3.59)	46 (6.74)^1^
13–17	1079 (19.75)	150 (21.99)
18–25	2650 (48.51)	185 (27.13)
≥ 25	1538 (28.15)	301 (44.13)
Age at ceasing smoking (years)		
Mean (SD)	52.1 (13.8)	52.3 (15.5)
< 40	186 (15.74)	27 (16.46)
< 50	259 (21.91)	33 (20.12)
< 60	361 (30.54)	47 (28.66)
≥ 60	376 (31.81)	57 (34.76)

Number may not added up to total due to missing data

^1^ P < 0.05

Table 2 showed baseline characteristics by sex and smoking status. In general, the baseline characteristics were similar between smokers and non-smokers in men, except for prevalence of residence status (79.72% vs. 70.73%) and drinkers (72.76% vs. 53.24%). In women, the prevalence of drinkers, hypertension, married, history of CVD was apparently higher in smokers, but smokers had lower percentage of overweight/obesity and middle school education.

**Table 2:** Baseline characteristics by sex and smokers

	Male			Female		
	Non-smoker (N = 2194)	Ex-smoker (N = 1182)	Smoker (N = 4281)	Non-smoker (N = 8362)	Ex-smoker (N = 164)	Smoker (N = 518)
Age(years)	60.13 (10.37)	62.50 (9.93)	58.93 (9.06)^1^	58.26 (10.33)	66.00 (11.18)	61.50 (10.13)^1^
Age group						
45–49	473 (21.56)	157 (13.28)	868 (20.28)^1^	2196 (26.26)	17 (10.37)	81 (15.64)^1^
50–59	699 (31.86)	344 (29.10)	1641 (38.33)	2935 (35.10)	41 (25.00)	158 (30.50)
60–69	605 (27.58)	391 (33.08)	1216 (28.40)	2059 (24.62)	41 (25.00)	161 (31.08)
70–79	326 (14.86)	233 (19.71)	479 (11.19)	872 (10.43)	47 (28.66)	102 (19.69)
≥ 80	91 (4.15)	57 (4.82)	77 (1.80)	300 (3.59)	18 (10.98)	16 (3.09)
Middle school education (%)	902 (41.11)	417 (35.28)	1689 (39.45)^1^	2003 (23.95)	16 (9.76)	74 (14.29)^1^
Rural (%)	1551 (70.73)	833 (70.47)	3412 (79.72)^1^	6574 (78.64)	133 (81.10)	435 (84.14)^1^
Married (%)	2155 (98.22)	1165 (98.56)	4210 (98.34)	8345 (99.80)	164 (100.00)	518 (100.00)
Drinker (%)	1168 (53.24)	884 (74.79)	3115 (72.76)^1^	1263 (15.10)	44 (26.83)	145 (27.99)^1^
Hypertension (%)	896 (40.84)	580 (49.07)	1652 (38.59)^1^	3495 (41.80)	85 (51.83)	244 (47.10)^1^
Dyslipidemia (%)	697 (31.77)	459 (38.83)	1276 (29.81)^1^	2673 (31.97)	66 (40.24)	178 (34.36)^1^
Diabetes (%)	172 (7.84)	131 (11.08)	229 (5.35)^1^	672 (8.04)	17 (10.37)	42 (8.11)
Overweight or obesity (%)	658 (29.99)	442 (37.39)	1001 (23.38)^1^	3100 (37.07)	67 (40.85)	161 (31.08)^1^
History of CVD (%)	282 (12.85)	233 (19.71)	402 (9.39)^1^	1222 (14.61)	58 (35.37)	114 (22.01)^1^

^1^ P < 0.05

Table 3 showed the effect of smoke on all-cause mortality and premature death. Generally, there was a 1.6-fold (HR = 1.58, 95%CI: 1.04–2.39) increase in the risk of premature death for smokers and 2.3-fold (HR = 2.25, 95%CI: 1.38, 3.66) for ex-smoker. Similar association was also found in the subgroup analysis by sex. The increase in the risk of premature death for smokers was 2.32 (95%CI: 1.27–4.26, adjusted analysis) in women. Additionally, there was a 1.4-fold (HR = 1.37, 95%CI: 1.02–1.83) increase in the risk of all-cause mortality for smokers and 1.8-fold (HR = 1.75, 95%CI: 1.24, 2.46) for ex-smoker. In the subgroup analysis, the risk of all-cause mortality in male smokers (HR = 1.67, 95%CI: 1.11–2.53) were found to be higher than female ex-smokers.

**Table 3:** Hazard Ratio of all-cause mortality or premature death among Smokers, as Compared with non-smokers in total and subgroup analysis ^1^

	Overall	Men	Women
All-cause mortality			
Ex-smoker			
Unadjusted	2.48 (1.85, 3.33)	1.95 (1.30, 2.93)	3.71 (1.95, 7.04)
Adjusted	1.75 (1.24, 2.46)	1.67 (1.11, 2.53)	1.82 (0.95, 3.48)
Smoker			
Unadjusted	1.31 (1.03, 1.65)	1.04 (0.73, 1.48)	1.97 (1.19, 3.26)
Adjusted	1.37 (1.02, 1.83)	1.27 (0.88, 1.83)	1.45 (0.87, 2.41)
Premature death ^2^			
Ex-smoker			
Unadjusted	2.78 (1.84, 4.18)	2.38 (1.36, 4.17)	2.34 (0.74, 7.46)
Adjusted	2.25 (1.38, 3.66)	2.08 (1.18, 3.69)	1.49 (0.46, 4.80)
Smoker			
Unadjusted	1.63 (1.18, 2.23)	1.23 (0.75, 2.02)	3.18 (1.76, 5.77)
Adjusted	1.58 (1.04, 2.39)	1.29 (0.77, 2.14)	2.32 (1.27, 4.26)

^1^ Adjusted for sex, age, highest level of education, current residence status, marital status, hypertension, dyslipidemia, diabetes, CVD, drink status, BMI

^2^ Premature death was defined as mortality before age 72.7 years in men and 76.9 years in women, which were the average life expectancies in China in 2011. However, age of death can only be estimated among CHALS 2013 follow up

Pack-years of smoking was another important factor for all-cause mortality and premature death. As shown in Table 4, pack-years of smoking ≥ 30 was associated with increased risk of premature mortality compared with non-smokers in total (HR = 1.59, 95%CI: 1.03, 2.43) and women (HR = 3.38, 95%CI: 1.22–9.38). Additionally, our results also revealed that there was a correlative trend between pack-years of smoking and premature death in total (P = 0.002) and women (P = 0.010). The association between pack-years of smoking and all-cause mortality was not statistically significant. This study also analyzed the effect of age at starting smoking and age at ceasing smoking on all-cause mortality and premature death and found no statistical significance (Supplement Table 1, Supplement Table 2).

**Table 4:** Hazard Ratio of all-cause mortality or premature death among different pack-years of smoking groups, as compared with non-smokers in total and subgroup analysis ^1^

	Overall	Men	Women
All-cause mortality			
0	1.00	1.00	1.00
< 15	0.93 (0.58, 1.49)	0.95 (0.57, 1.60)	0.66 (0.21, 2.06)
15–30	1.05 (0.66, 1.65)	0.98 (0.60, 1.61)	0.92 (0.23, 3.73)
≥ 30	1.27 (0.92, 1.75)	1.12 (0.79, 1.58)	2.22 (0.90, 5.46)
P for trend ^2^	0.349	0.245	0.037
Premature death			
0	1.00	1.00	1.00
< 15	1.22 (0.68, 2.17)	1.30 (0.69, 2.44)	0.43 (0.06, 3.08)
15–30	1.34 (0.76, 2.36)	1.26 (0.69, 2.30)	0.98 (0.14, 7.06)
≥ 30	1.59 (1.03, 2.43)	1.41 (0.89, 2.24)	3.38 (1.22, 9.38)
P for trend ^2^	0.002	0.185	0.010

^1^ Adjusted for sex, age, highest level of education, current residence status, marital status, hypertension, dyslipidemia, diabetes, CVD, drink status, BMI

^2^ P values for trend were calculated using the quartile median values

## Discussion

In this cohort study, the prevalence of tobacco smoke remained substantial in China, particularly among males. Furthermore, tobacco smoker was associated with increased risk of all-cause mortality in total and premature death in total and women. Ex-smoker was associated with increased risk of all-cause mortality in total and men and premature death in total and men. The pack-years of smoking were also associated with increased risk of premature death in both the overall sample and the women.

Previous studies had demonstrated a significant decrease in tobacco smoke prevalence in several high-income countries, particularly among men [14–16]. For example, Australia has achieved notable success in tobacco control, as evidenced by previous data indicating that the prevalence of current and past smokers was 7.7% and 34.1% respectively, with similar numbers observed among both genders [16]. Additionally, a pooled analysis of 21 Asian cohort studies reported a total prevalence of ever smoking at 65.1% among men and 7.1% among women [15]. In the present study, we found that prevalence of tobacco use remained high in China, and prevalence in men was considerably higher than women. Despite the documented decline in global tobacco consumption across various studies, it was noteworthy that the prevalence of tobacco smoke was still on the rise in developing countries including China.

The impact of tobacco smoke on all-cause mortality had been comprehensively documented through successive birth cohort studies conducted in UK and the US. These studies have revealed RRs for all-cause mortality ranging from approximately 1.4 to 1.8 in the 1960s to 2.1 to 2.3 in the 1980s, and further reaching 2.8 to 3.0 in 2000s among smokers versus non-smokers [5, 6, 17–19]. The incremental progression in RRs had been attributed to the earlier initiation of smoking and the heightened intensity of tobacco exposure among successive birth cohorts. Our findings revealed a significant association between tobacco smoker/ex-smoker and all-cause mortality, while such a correlation was also observed between males and ex-smokers. Because this study was an ongoing study which time interval between baseline and follow up survey was only two years, leading to a constrained sample size of recorded deaths. Additionally, it was worth noting that existing evidence suggested a higher prevalence of multiple risk factors among men compared to women [20]. Moreover, CHARLS did not encompass the collection of data pertaining to cause-specific deaths. As a result, we were constrained to utilize all-cause mortality as the primary outcome, potentially introducing the element of competing risks of death. We also found smoke was an important cause for premature death. In the subgroup analysis, our findings revealed a significant association between tobacco smoke and premature death exclusively among female. This outcome was similar to the results of a systematic analysis highlighted tobacco smoke as a significant driver of premature death [20]. In the US, smoke remained leading cause of premature death. A report had projected that a staggering 5.6 million Americans under the age of 18, currently alive, would face premature death due to smoke-related diseases, albeit that the prevalence of cigarette smoke had fallen over the past five decades since the 1964 report from 42 to 18% in 2012 [21].

Many epidemiology studies only estimated the impact of current smoking, but not estimated the effect of smoke intensity and duration. Our results found smoke intensity was an important factor for premature death. In addition, a discernible trend in the association between the number of packyears and premature death was also identified. These findings were consistent with observations from several cohort studies conducted in Western countries. For example, results from a cohort study conducted in Netherland showed that a higher baseline number of pack years was associated with an increased risk of all-cause and various cancer-related mortality [22]. An additional cohort study in the United States revealed a notable increase in the risk of both all-cause mortality and breast cancer-specific mortality among individuals with a smoking history exceeding 20 pack-years [23]. Our results suggested the impact of tobacco smoke and pack-years of smoking on death were different by sex, the effect was more pronounced in women. The disparity in effects between genders might be partly explained by the different effect of tobacco smoke on specific cancer incidence. In detail, a cohort study conducted in the US found that females were susceptible to develop lung cancer than males [24]. Another a large prospective study in the US, known as Nurse’s health study, reported a positive correlation between breast cancer incidence and a higher pack-years of smoking [23]. Unfortunately, our study lacked the information about cause of death and sample size of death was limited to support us to do similar analysis. Consequently, further researches were needed to confirm this finding of sex difference on the effect of tobacco smoke and pack-years of smoking.

Our study had several strengths and limitations. Because of the longitudinal nature of the data, we could reveal linkages between smoke and deaths. Smoking status information was collected at baseline rather than retrospectively, ensuring that this study suffered less potential bias. Some limitations should be noticed: Firstly, this study was an ongoing study, the sample size of deaths was limited. Secondly, only data of 2013 follow-up survey was selected, because we needed to analyze the effect of smoking status on premature death and date of death could only be estimated among the data of 2013 follow-up survey. Thirdly, we did not collect the information about cause of death which limit our further analysis to reveal the cause of different effect of smoking status on mortality among men and women.

## Conclusion

Our results revealed a high prevalence of smokers within the Chinese population, particularly among men, and confirmed the detrimental impact of smoking on all-cause mortality and premature death from a contemporary and nationally representative data from China. We had also identified a correlation between pack-years of smoking and premature death. These results underscore the urgent need for more stringent tobacco control measures in China, with the aim of reducing the prevalence of smoking.

### Electronic supplementary material

Below is the link to the electronic supplementary material.

Supplementary Material 1

## Data Availability

All datasets are available from the China Health and Retirement Longitudinal Study (CHARLS) database (https://charls.charlsdata.com/pages/data/111/en.html, 2011 CHARLS Wave 1, 2013 CHARLS Wave 2). Researchers who want to use these data can register accounts via the website.

## Abbreviations

CHARLS: China Health and Retirement Longitudinal Study
CVD: cardiovascular disease
DBP: diastolic blood pressure
HR: Hazard Ratio
PPS: probability proportional to scale
SBP: systolic blood pressure
