# Supplementary material for: Tobacco smoke and all-cause mortality and premature death in China: a cohort study
Source: BMC Public Health. 2023 Dec 12;23:2486. doi: 10.1186/s12889-023-17421-w (PMC10714570; doi:10.1186/s12889-023-17421-w)
Supplement: Supplementary file 1 — Supplementary Material 1 [file 12889_2023_17421_MOESM1_ESM.docx]

**Supplementary Table 1** Hazard Ratio of all-cause mortality or premature death among different age at starting smoking groups in total and subgroup analysis ^1^

|  | Overall | Men | Women |
| --- | --- | --- | --- |
| All-cause mortality | | | |
| <13 | 1.00 | 1.00 | 1.00 |
| 13-17 | 1.41 (0.69, 2.89) | 1.71 (0.72, 4.04) | 0.66 (0.16, 2.66) |
| 18-25 | 1.08 (0.54, 2.17) | 1.19 (0.51, 2.75) | 0.79 (0.20, 3.14) |
| ≥25 | 0.63 (0.30, 1.32) | 0.62 (0.25, 1.53) | 0.78 (0.21, 2.93) |
| Premature death | | | |
| <13 | 1.00 | 1.00 | 1.00 |
| 13-17 | 2.12 (0.64, 6.97) | 4.71 (0.64, 34.84) | 1.04 (0.19, 5.75) |
| 18-25 | 1.30 (0.40, 4.24) | 2.85 (0.39, 20.85) | 0.62 (0.10, 3.68) |
| 0.60≥25 | 1.10 (0.33, 3.69) | 2.49 (0.33, 18.69) | 0.60 (0.11, 3.43) |

^1^ Adjusted for sex, age, highest level of education, current residence status, marital status, hypertension, dyslipidemia, diabetes, CVD, drink status, BMI.

**Supplementary Table 2** Hazard Ratio of all-cause mortality or premature death among different age at ceasing smoking groups in total and subgroup analysis ^1^

|  | Overall | Men | Women |
| --- | --- | --- | --- |
| All-cause mortality | | | |
| <40 | 1.00 | 1.00 | 1.00 |
| <50 | 1.86 (0.48, 7.21) | 1.91 (0.37, 9.89) | 8.78 (0.30, 256.84) |
| <60 | 2.47 (0.72, 8.46) | 3.14 (0.71, 13.86) | 1.32 (0.05, 35.71) |
| ≥60 | 2.45 (0.71, 8.45) | 3.96 (0.88, 17.89) | 0.24 (0.01, 4.33) |
| Premature death | | | |
| <40 | 1.00 | 1.00 | 1.00 |
| <50 | 2.71 (0.30, 24.34) | 2.73 (0.30, 24.46) | - |
| <60 | 8.22 (1.05, 64.13) | 7.92 (1.00, 62.74) | - |
| ≥60 | 9.71 (1.06, 89.26) | 9.35 (0.96, 90.81) | - |

^1^ Adjusted for sex, age, highest level of education, current residence status, marital status, hypertension, dyslipidemia, diabetes, CVD, drink status, BMI.
